# Hypoxic tumor cell-derived small extracellular vesicle miR-152-3p promotes cervical cancer radioresistance through KLF15 protein

**DOI:** 10.1186/s13014-023-02369-3

**Published:** 2023-11-07

**Authors:** Junying Zhou, Ningjing Lei, Wanjia Tian, Ruixia Guo, Feng Gao, Hanlin Fu, Jing Zhang, Shiliang Dong, Mengyu Chen, Qian Ma, Yong Li, Lei Chang

**Affiliations:** 1 Department of Obstetrics and Gynecology, The First Affiliated Hospital of Zhengzhou University, No.1 Jianshe East Road, Zhengzhou, 450052 Henan China; 2 School of Basic Medical Sciences, Zhengzhou University, Zhengzhou, Henan China; 3 Department of Neuroimmunology, Henan Institute of Medical and Pharmaceutical Sciences, Zhengzhou University, Zhengzhou, Henan China; 4 Henan Engineering Technology Research Center for Accurate Diagnosis Neuroimmunity, Zhengzhou, Henan China; 5 Department of Radiation Oncology, The First Affiliated Hospital of Zhengzhou University, Zhengzhou, Henan China; 6 Level 2, Research and Education Centre, Cancer Care Centre, St George Hospital, 4-10 South St, Kogarah, NSW 2217 Australia; 7 St George and Sutherland Clinical Campuses, School of Clinical Medicine, UNSW Sydney, Kensington, NSW Australia

**Keywords:** Cervical cancer, Radioresistance, Hypoxia, Extracellular vesicle, miR-152-3p

## Abstract

**Background:**

Radiotherapy is widely used in treating cervical cancer patients, however, radioresistance unavoidably occurs and seriously affects the treatment effect. It is well known that hypoxia plays an important role in promoting radioresistance in tumor microenvironment, yet our understanding of the effect of small extracellular vesicles miRNA on cervical cancer radiosensitivity in hypoxic environment is still limited.

**Methods:**

Small extracellular vesicles extracted from hypoxic and normoxic cultured cervical cancer cells were evaluated for their effects on radioresistance. miR-152-3p was found to be a potential effector in hypoxia-derived extracellular vesicles by searching the GEO database. Its downstream substrate was confirmed by double luciferase report, which was KLF15. The role of miR-152-3p and KLF15 in regulating cervical cancer radioresistance was detected by cell activity assays. The findings were confirmed in vivo by animal models. The expression of miR-152-3p was quantified by qRT-PCR and its prognostic significance was evaluated.

**Results:**

Hypoxic environment promoted the secretion of small extracellular vesicles, and reduced the apoptosis and DNA damage caused by radiation, accompanied by increased expression of small extracellular vesicles miR-152-3p from hypoxic cervical cancer cells. Furthermore, small extracellular vesicles miR-152-3p promoted Hela xenograft growth and reduced the radiosensitivity *vivo*. Mechanism studies revealed that KLF15 protein was the downstream target of miR-152-3p in regulating radioresistance.

**Conclusion:**

Our findings suggest that small extracellular vesicles miR-152-3p affects the therapeutic effect of radiotherapy and holds potential as a biomarker or therapeutic target for cervical cancer prognosis and improving radiotherapy.

**Supplementary Information:**

The online version contains supplementary material available at 10.1186/s13014-023-02369-3.

## Introduction

Cervical cancer (CC) brings a huge disease burden on women due to its high morbidity and mortality all over the world [1]. Currently, various therapeutic strategies including surgery, radiotherapy (RT), chemotherapy, and immunotherapy are applied in treating CC. For locally advanced CC, simultaneous RT and chemotherapy have shown promising effects [2, 3]. However, radioresistance has become one of the main factors limiting the efficacy of RT [4]. Therefore, enhancing the effectiveness of RT for the treatment of CC patients requires a deeper knowledge of the mechanisms underlying radioresistance.

It is well known that hypoxia is associated with radioresistance since free radicals come from oxygen and are necessary to kill tumor cells for ionizing radiation (IR) [5, 6]. In addition, the hypoxic environment also produces other biological changes in tumors. For instance, decreased cell oxygen content in the tumor microenvironment (TME) interferes with radiation-induced cell apoptosis, DNA damage and other biological processes that are beneficial to curative effects [7]. Therefore, whether and how the hypoxia environment affects the efficacy of RT requires in-depth research.

Most of the research on the interaction between hypoxia and radioresistance focuses on hypoxic tumor cells [8, 9], but the communication between cells in the TME is not well investigated. Areas of hypoxia are usually distributed throughout the surviving tumor mass. Extracellular vesicles (EVs) are a general term for a variety of membrane-enclosed nanosized vesicles carrying cell-derived cargo. These vesicles range in diameter from 50 to 1000 nm and are released from all types of cells under normal physiological conditions and pathological processes [10, 11]. Two prominent kinds of EVs that have been long recognized are small EVs (sEV, i.e. exosomes, 40–150 nm in diameter) and large EVs (lEV, i.e. microvesicles, 150–1000 nm in diameter) [12–14]. In this study, all exosomes quoted from references are referred to as EVs because EVs constitute a diverse population of vesicles, according to the standards of the International Society for Extracellular Vesicles (ISEV) [15]. EVs released by hypoxic tumor cells promote intercellular communication and thus play various roles in different biological processes [16]. EVs usually play their biological roles by wrapping microRNA, mRNA or protein in a cargo [17, 18]. The stable miRNA encapsulated by EVs was reported to be used as an effector molecule, conferring biological changes induced by hypoxia, such as radioresistance [19].

From the perspective of the hypoxic environment, EVs in the TME may provide a novel outlook for the research of CC radioresistance. In the study, we found that EVs induced by hypoxia enhanced CC radioresistance by reducing apoptosis and DNA damage caused by radiation. In addition, we also for the first time demonstrated sEV miR-152-3p was highly expressed in hypoxic CC cells, which could be transferred to normoxic cells and affect radioresistance by targeting its downstream substrate Kruppel-like factor 15 (KLF15) in vitro. The regulatory effect of miR-152-3p on the progression of prostate cancer and liver cancer was studied previously. It was reported that miR-152-3p inhibited the proliferation of prostate cancer cells by targeting KLF4 [20]. Another study showed that miR-152-3p inhibited the proliferation and promoted the apoptosis of hepatocellular carcinoma cells by repressing roundabout guidance receptor 1 [21]. However, miR-152-3p has not been reported in the regulatory mechanism of cervical cancer progression. So we detected that increased expression of sEV miR-152-3p reduced the killing effect of irradiation on tumor in a xenograft tumor experiment in vivo, associated with inhibiting cell apoptosisin cervical cancer cell lines SiHa and Hela. Furthermore, we showed that the high expression of plasma sEV miR-152-3p was associated with the risk of recurrence in clinical CC patients. Our findings suggest that sEV miR-152-3p is a useful biomarker for predicting CC radioresistance as well as a potential therapeutic target for CC treatment.

## Methods

### Patients and samples

Our study recruited 40 patients with CC who underwent surgery from 2017 to 2019. Paraffin-embedded tumor tissues and adjacent normal tissues (> 5 cm from tumor tissue) were collected during the surgery for detecting the level of miR-152-3p. In addition, blood (10 mL/tube) of 20 CC patients who did not relapse after RT and 20 CC patients who relapsed after RT were collected for prognostic analysis. Patients included in this study were followed up every 3 months until death or April 2021. All cases were confirmed by experienced pathologists, and none of them had received chemotherapy or RT before surgery. Prior to the study, the written informed consent of all patients was obtained. The study protocol was approved by the Ethics Committee (No.2022-ky-1371-002) and was based on the ethical principles of the Declaration of Helsinki for medical research involving human beings.

### Cell lines and cell culture

Normal cervical epithelial cell line (Ect1/E6E7) was purchased from BeiNa biological company (China) and CC cell lines (SiHa, C33A and Hela) were purchased from Procell biological company (China). Normal cervical epithelial cell line was cultured with DMEM medium (Gibco, MA, USA) and CC cell lines (SiHa, C33A and Hela) were cultured with MEM medium (Gibco, MA, USA). All media contain 10% fetal bovine serum (FBS) (PAA, Bentley, Australia) and 1% penicillin–streptomycin (Procell, Wuhan, China). All cell lines were kept at 37 °C in a humidified environment with indicated CO_2_ concentrations.

### Hypoxia treatment

In this study, the normal oxygen cells were cultured in an incubator at 37 °C with 5% CO_2_, while the hypoxic cells were cultured in another incubator at 37 °C with 1% O_2_, 5% CO_2_ and 94% N_2_. Hypoxic cells were confirmed by western blot analysis to detect levels of hypoxia-inducible factor 1α (HIF-1α) in cultured cells [22, 23].

### Cell transfection

KLF15 overexpression plasmid and its negative control, miR-152-3p mimic or inhibitor and their negative controls were purchased from GenePharma (Shanghai, China). The stable knockdown of KLF15 was performed with lentiviral short hairpin RNA targeting KLF15 (sh-KLF15), and its negative control were produced by Genechem (Shanghai, China). The transfection was constructed with Lipofectamine 2000 (Invitrogen, Ma, USA) according to the manufacturer's protocols. The information about plasmids is listed in Additional file 1: Table S1.

### Quantitative real-time PCR (qRT-PCR)

Total RNA was extracted using the High Pure RNA Isolation Kit (Ambion, USA) according to the manufacturer’s instruction. The qRT-PCR was performed using our previously established method [24]. GAPDH and U6 were used as internal references, and the relative gene expression was calculated using the 2^−ΔΔCt^ method. The information about plasmids is listed in Additional file 1: Table S2.

### sEV isolation from cell lines

After the cells (SiHa and Hela) were incubated in 20 mL fresh sEV-depleted medium for 48 h, the supernatant from each cell line was collected. The supernatant was centrifuged at 2000 *g* for 20 min at 4 °C to remove dead cells and followed by 10,000 *g* for 45 min. The samples were then filtered using a sterile filter (0.22 μm, Millipore, USA). Centrifugation was conducted at 100,000 *g* for 80 min, and the supernatant was discarded, and then resuspended with PBS. After the last centrifugation (100,000 *g*, 80 min), the sample was dissolved with PBS and added with RNase I. Purified EVs were collected and stored at − 80 °C.

### sEV isolation from blood sample

Whole blood (10 mL/tube) from patients was collected in tubes containing EDTA, and the plasma was separated by centrifuging the tubes at 3000 *g* for 15 min at 4 °C. Plasma samples were mixed with ExoQuick ULTRA kit (System Biosciences, Palo Alto, CA, USA) according to the manufacturer's protocol, co-incubated at 4 °C for 30 min and then centrifuged at 3000 *g* for 10 min. After discarding the supernatant, 200 μL of ExoQuick ULTRA buffer was added for resuspension and a further 200 μL of buffer A was added for resuspension. The suspension was added to the resin column and purified by centrifugation at 1000 *g* for 2 min. Purified EVs were collected and stored at − 80 °C.

### sEV identification

TEM was used to determine the morphology of sEV samples. The NanoSight NS300 system (Malvern Instruments, Malvern, UK) was used to find the size distribution and concentrations of sEVs. A detailed description of the method is included in Additional file 2.

### Western blot (WB) analysis

Total protein from sEVs or cell lines was obtained using the RIPA lysis buffer containing 1 × Halt™ protease and phosphatase inhibitor cocktail (Beyotime, China) and their concentrations were then assessed using a BCA assay (Thermo Fisher Scientific, USA). The western blotting analysis was carried out as previously described [24]. Antibodies used are listed in Additional file 1: Table S3. CD63, CD81, Alix and Calnexin were used to identify sEV characterization.

### External miRNAs inhibition of EVs

To eliminate the interference of the external EV miRNAs of EVs, RNase R (5 µg/mL) was added to the EVs suspension from cell suspension, incubated for 15 min, and the level of EV miR-152-3p was detected by qRT-PCR. 0.1% Triton X-100 and RNase R were added to the EV suspension for 30 min, and the level of miR-152-3p was detected by qRT-PCR to eliminate interference from nonspecific precipitates.

### Cell proliferation

Proliferating capacity of transfected CC cell lines SiHa and Hela, and the control cell line Ect1/E6E7 was detected by the Cell Counting Kit-8 (DOJINDO, Japan) according to the instruction. A detailed description of the method is included in Additional file 2.

### Immunofluorometric assay

5 × 10^4^ cells were inoculated into 24-well plates with glass cover-slips and irradiated after 24 h. After 4 h, the cells were fixed with 4% paraformaldehyde for 30 min, and infiltrated with 0.1% Triton X-100 for 2 h, respectively. After sealing with 5% BSA for 90 min, washing with PBS and incubation overnight (o/n) with anti-γ-H2AX primary antibody (1:400; Abcam, USA). The primary antibody was removed with PBS, followed by incubation with Alexa fluor 555 conjugated secondary antibody (Beyotime, China) for 90 min at room temperature. DAPI was used to stain the nuclei of cells and then visualized under the confocal microscope (Leica, Germany).

### Flow cytometry for cell apoptosis detection

SiHa and Hela cells were cultured in 6-well plates for 24 h and then irradiated at a dose of 4 Gy [25]. For apoptosis assays, cells were collected after centrifugation and resuspended in the binding buffer, then stained using Annexin V and propidium iodide (PI) (BestBio, China). At last, measurements were made using flow cytometry (Beckman Coulter, USA). The results were subsequently calculated with FlowJo software (Tree Star Inc., Ashland, OR, USA).

### Invasion assay

Commercial Matrigel and transwell chambers were used to measure the incasiveness of the transfected CC cells (BD Bioscience, CA, US). A detailed description of the method is included in Additional file 2.

### Mouse xenograft experiment

Seventy female BALB/c nude mice (5 weeks old) were obtained from Charles River Laboratories (China). Briefly, Hela cells (2 × 10^6^) were collected and subcutaneously injected into the right lower abdomen of the mice. The tumor size was measured every 5 days after injection. Tumor volume was calculated: volume = (width^2^ × length)/2. When the tumor volume reached 50 mm^3^, 10 μg/50 μL EVs or 50 μL PBS was injected into the tumor tissue once a day for 5 days. In addition, the tumor was irradiated locally at a dose of 4 Gy per day for 4 days after the second day of EVs injection and other part of animal was shielded with lead during irradiation. In addition, in the same xenograft tumor model, PBS or miR-152-3p mimics was injected into nude mice, respectively. Two weeks after irradiation, the mice were euthanized and tumor xenograft tissues were collected for further analysis. The animal treatment was grouped as follows: PBS + IR (n = 6), N-EV + IR (n = 6) and H-EV + IR (n = 6); NC + IR (n = 6) and miR-152-3p + IR (n = 6).

### Immunohistochemistry (IHC)

At the end of the experiment, the fresh tumor xenograft tissue was quickly frozen or fixed by 4% formalin, then embedded in paraffin and sliced for IHC and TUNEL staining, respectively. Hematoxylin and eosin (H&E)-stained sections was used to determine the tumor structure. A detailed description of the method and assessment of immunostaining was included in Additional file 2. Antibodies used were listed in Additional file 1: Table S3. The assessment criteria were as previously reported [26].

### TUNEL assay for apoptotic cells in vivo

To evaluate apoptosis in animal xenograft tumors with different treatments, TUNEL assay was performed in paraffin-embedded sections using a Tunel analysis kit (Roche, Switzerland) according to the manufacturer's protocol. Images were recorded by a light microscope (Nexcope NE900, China) (Magnification, × 200). The staining results were assessed using above-mentioned method.

### Dual luciferase reporter assay

The miR-152-3p target binding site on KLF15 was determined by Starbase online software (ENCORI). The predicted miR-152-3p binding region was amplified by PCR and cloned into the pMIR reporter plasmid in either the wild-type or mutant KLF15 3'UTR. After inoculation of SiHa and Hela cells into 24-well plates and incubation for 24 h, two luciferase reporter plasmids containing KLF15 and miR-152-3p simulant were co-transfected into cells. After 48 h, the Dual Luciferase Assay Kit (Promega, USA) was used to detect luciferase activity. Three independent experiments were performed.

### Ribonucleoprotein immunoprecipitation (RIP) assay

To find out the downstream target protein of miR-152-3p, 5 × 10^7^ cultured cells treated with miR-152-3p mimics and control plasmids were collected and lysed, and the lysates were incubated with anti-AGO G magnetic beads (Thermo Scientific, Massachusetts, USA) o/n. The magnetic beads were collected, washed with RIPA buffer and resuspended in 50 mmol/L Tris HCl at pH 7.0. Finally, RNA was isolated and enriched from magnetic beads and analyzed quantitatively by qRT-PCR.

### Statistical analysis

Date were analyzed using SPSS 22.0 and GraphPad prism 9 software and shown as the mean ± standard deviation. Unpaired student’s *t* test was used to analyze the differences between the two groups of data, and one-way analysis of variance (ANOVA) was used to analyze the differences of data in three or more groups. *P < 0.05, **P < 0.01, ***P < 0.001.

## Results

### Hypoxic CC cell-derived sEVs reduce cell damage induced by IR and enhance radioresistance

**Fig. 1:**
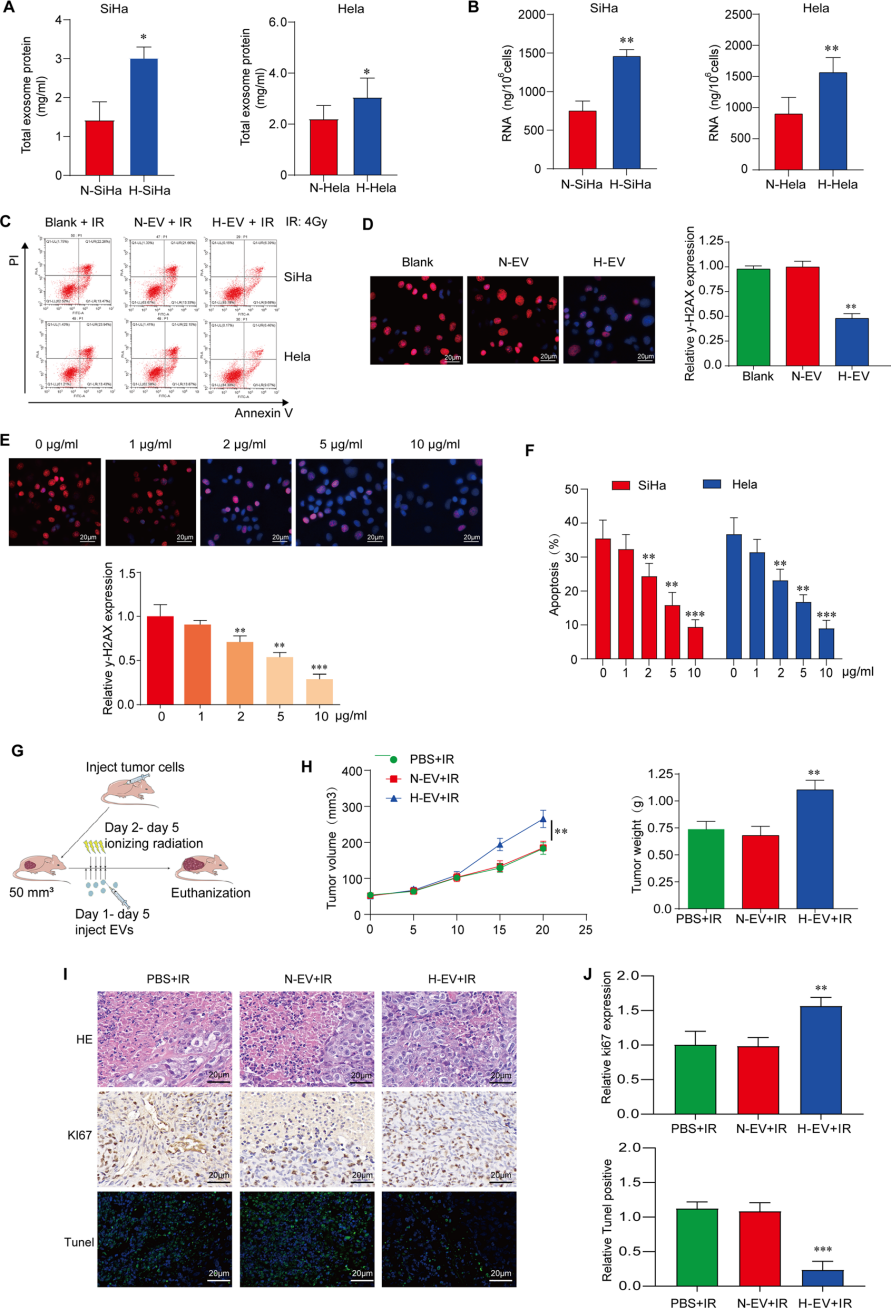
Hypoxic tumor cell-derived sEVs alleviate cell damage induced by IR and enhance radioresistance. **A** The expression of total protein in sEVs derived from hypoxic and normoxic CC cells was detected by BCA method. *P < 0.05 versus normoxic tumor cells. **B** Nano was used to detect the total RNA content in sEVs derived from hypoxic and normoxic tumor cells. **P < 0.01 versus normoxic tumor cells. **C** SiHa and Hela CC cells were co-cultured with PBS, NE-sEVs, HE-sEVs, tumor cell apoptosis was measured after 4 Gy radiation treatment by flow cytometry. **D** Expression of γ-H2AX protein in blank control cells, HE-sEVs or NE-sEVs-treated cells after 4 Gy radiation treatment was detected by Immunofluorometric assay. **P < 0.01 versus normoxic tumor cells with PBS. **E** Expression of γ-H2AX protein after different doses of HE-sEV treatment was detected by Immunofluorometric assay. *P < 0.05 and **P < 0.01 versus normoxic tumor cells with PBS. **F** The apoptotic cells treated with different doses of HE-sEVs in SiHa and Hela CC cells was detected and quantitatively analyzed by flow cytometry. *P < 0.05 and **P < 0.01 versus normoxic tumor cells with PBS. **G** The flow chart of animal study. **H** The tumor volume and weight in mice after different treatments with PBS + IR, NE-sEVs + IR, HE-sEVs + IR was measured (n = 6 in each treatment group). **P < 0.01 versus PBS + IR group and HE-sEVs + IR group. **I** The representative images of HE, Ki67 and TUNEL staining in PBS + IR, NE-sEVs + IR, HE-sEVs + IR groups. In HE, less damage is seen in HE-sEVs treatment; in Ki67 staining, brown indicates positive stating while blue indicates nuclear; in TUNEL staining, green indicates positive staining. Scale bar: 20 μm. All irradiation treatment was performed with a single 4 Gy. **J** Right panel shows the quantification of Ki67-positive and TUNEL-positive cells in PBS + IR, NE-sEVs + IR, HE-sEVs + IR groups. **P < 0.01 and ***P < 0.001 versus PBS + IR group and NE-sEVs + IR group

To study the effect of sEVs derived from the hypoxia environment on CC radiation response, we first isolated sEVs from supernatants secreted by CC cells treated in a hypoxia environment (HE) or a normoxia environment (NE). The isolated sEVs were detected by transmission electron microscopy (TEM), nanoparticle tracking analysis (NTA) and western blot analysis. We discovered that 150 nm was the particle size that was most widely disseminated from SiHa and Hela cell lines (Additional file 1: Fig. S1A). In addition, NTA also demonstrated that hypoxia increased the number of sEVs secreted by both Hela and SiHa cells in HE compared to cells treated in NE (Additional file 1: Fig. S1B). TEM results showed different oxygen concentrations did not change the morphology of sEVs from HE and NE conditions (Additional file 1: Fig. S1C). The expression of sEV markers CD63, CD81 and Alix were considerably enhanced in HE-treated SiHa and Hela cells, and the negative control marker, Calnexin, showed deletion of expression, which verified the identity of sEVs (Additional file 1: Fig. S1D, E). Furthermore, we showed total sEV proteins and RNAs were increased in HE compared to those observed in NE (Fig. 1A, B), indicating that HE led to an increased sEV secretion. To confirm the accurate construction of the hypoxia model, we tested HIF-1α by western blot analysis in our model and demonstrated increased expression of HIF-1α in HE model compared to NE one (Additional file 1: Fig. S1F). Finally, we used PKH67 to label and detect sEVs and showed that the labelled sEVs could successfully enter CC cells (Additional file 1: Fig. S1G).

We then co-cultured SiHa and Hela CC cells with HE-sEVs and NE-sEVs to find the difference of effects of sEVs from two models on CC radiation response. Our results indicated that HE-sEVs produced an anti-radiation effect and significantly reduced cell apoptosis after IR (Fig. 1C), while the expression of DNA damage protein γ-H2AX in two CC cell lines after HE-sEV treatment was significantly lower than that in untreated group or NE-sEV treatment group. And this protective effect was more obvious with the increase of sEV concentration (Fig. 1D, E). Cell apoptosis assay further confirmed that HE-sEV treatment significantly reduced SiHa and Hela cell death compared with that from NE-sEV treatment (Fig. 1F).

To further investigate the radioresistant effects of HE-sEVs in vivo, we established tumor xenograft mouse models and injected 10 μg/50 μL HE-sEVs or NE-sEVs or 50 μL PBS as negative control into the tumor sites when tumor volume reached about 50 mm^3^ (Fig. 1G). After injection, we found that the growth of tumor volume and weight in HE-sEVs injection group was significantly increased compared with NE-sEVs injection group and PBS control group after IR, respectively (Fig. 1H). In tumor xenograft examination after RT, we found increased expression of Ki67 (1.58 ± 0.14) and reduced TUNEL-positive cells (0.23 ± 0.13) in HE-sEVs + IR treatment group in comparison with that in NE-sEVs + IR group or PBS + IR group with Ki67 (0.98 ± 0.12), (0.99 ± 0.21) and TUNEL-positive cells (1.06 ± 0.14) and (1.12 ± 0.10), respectively, indicating that HE-sEVs + IR treatment significantly reduced radiation-induced damage by increasing the cell proliferation and inhibiting apoptosis of the two CC cells ( Fig. 1I, J). These results demonstrate that sEVs derived from hypoxic tumor cells reduce IR-induced cell damage and promote CC radioresistance in vitro and in vivo.

### MiR-152-3p affects the radiosensitivity of CC cells

**Fig. 2:**
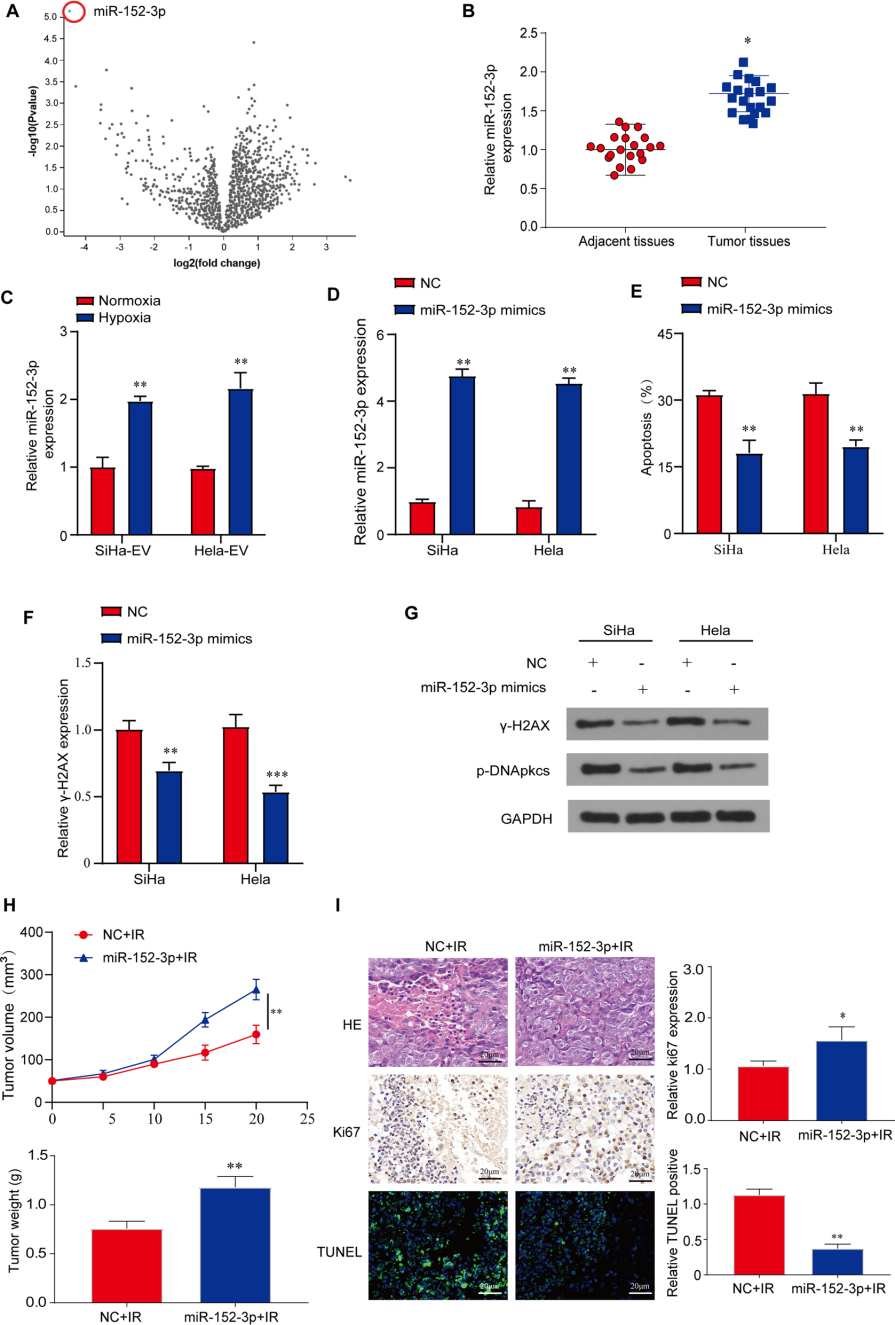
The role of miR-152-3p in CC radiation response. **A** The differentially expressed miRNAs from GSE81137 database were shown by volcanic map. **B** The level of miR-152-3p expression in cervical tumor tissues compared to adjacent tissues was examined by qRT-PCR, n = 20 in each group. *P < 0.05 versus adjacent tissues. **C** The significant difference of sEVs miR-152-3p expression was found in SiHa and Hela cells with different oxygen contents (normoxia and hypoxia) by qRT-PCR. **P < 0.01 versus CC cells with normoxia. **D** A significant difference of miR-152-3p expression was found after miR-152-3p mimic treatment on SiHa and Hela CC cells by qRT-PCR, **P < 0.01 versus negative control CC cells. **E** Flow cytometry was conducted to detect the effect of miR-152-3p mimics on radiation-induced apoptosis in SiHa and Hela cells. **P < 0.01 versus negative control CC cells. **F** The significant difference of expression of the DNA damage markers γ-H2AX was shown by immunofluorometric assay after miR-152-3p mimic treatment on SiHa and Hela CC cells. **P < 0.01 and ***P < 0.001 versus negative control CC cells. **G** The reduced expression of γ-H2AX and p-DNApkcs was shown by western blot in SiHa and Hela cells treated with miR-152-3p mimic or negative control (NC). **H** Hela cells transfected with miR-152-3p mimics and negative control (NC) plasmid were injected into nude mice, respectively, tumor volume and weight after irradiation were measured, n = 6 in each group. **P < 0.01 versus negative control CC cells. **I** Representative images of HE, Ki67 and TUNEL staining with NC + IR and miR-152-3p + IR, scale bar: 20 μm. All irradiation treatment doses used were 4 Gy. Right panel shows the quantification of Ki67 and TUNEL positive cells in two treatment groups. *P < 0.05 and **P < 0.01 versus negative control CC cells

To find out if the expression level of miRNAs is associated with CC RT, we first analyzed the NCBI database (GSE81137) and found that miR-152-3p was significantly upregulated in CC tissues (Fig. 2A). We then examined miR-152-3p expression in CC tissues using our existing tissue archive and found higher expression level of miR-152-3p was detected in CC tissues compared to the adjacent normal tissues (Fig. 2B). Furthermore, we also found the expression of miR-152-3p in HE-sEVs is higher than that in NE-sEVs derived from both SiHa and Hela cells (Fig. 2C). To explore whether miR-152-3p affects radiosensitivity of CC cells, we transfected miR-152-3p mimics into SiHa and Hela cells (Fig. 2D). Our results showed that IR-induced apoptosis was significantly reduced after the overexpression of miR-152-3p (Fig. 2E). Meanwhile, miR-152-3p mimics significantly reduced the DNA damage of the transfected CC cells compared to control cells after irradiation (Fig. 2F, G). To further investigate the role of tumorigenicity of miR-152-3p in CC radiation, we conducted a subcutaneous tumor xenograft experiment. Similarly, we observed that injection of miR-152-3p mimics significantly promoted tumor growth and increased tumor weight in the IR group compared with control group (Fig. 2H). In addition, overexpression of miR-152-3p protected IR-induced cell damage by increasing cell proliferation (Ki67 positive cells) and reducing cell apoptosis (TUNEL-positive cells) compared with negative control (Fig. 2I). These results show that miR-152-3p plays an important role in the sensitivity of RT for CC. It reduces the apoptosis and DNA damage of CC cells caused by IR treatment, therefore enhancing the radiation tolerance of CC cells.

### Hypoxia cell-derived sEV miR-152-3p is an important cargo molecule mediating CC radioresistance

**Fig. 3:**
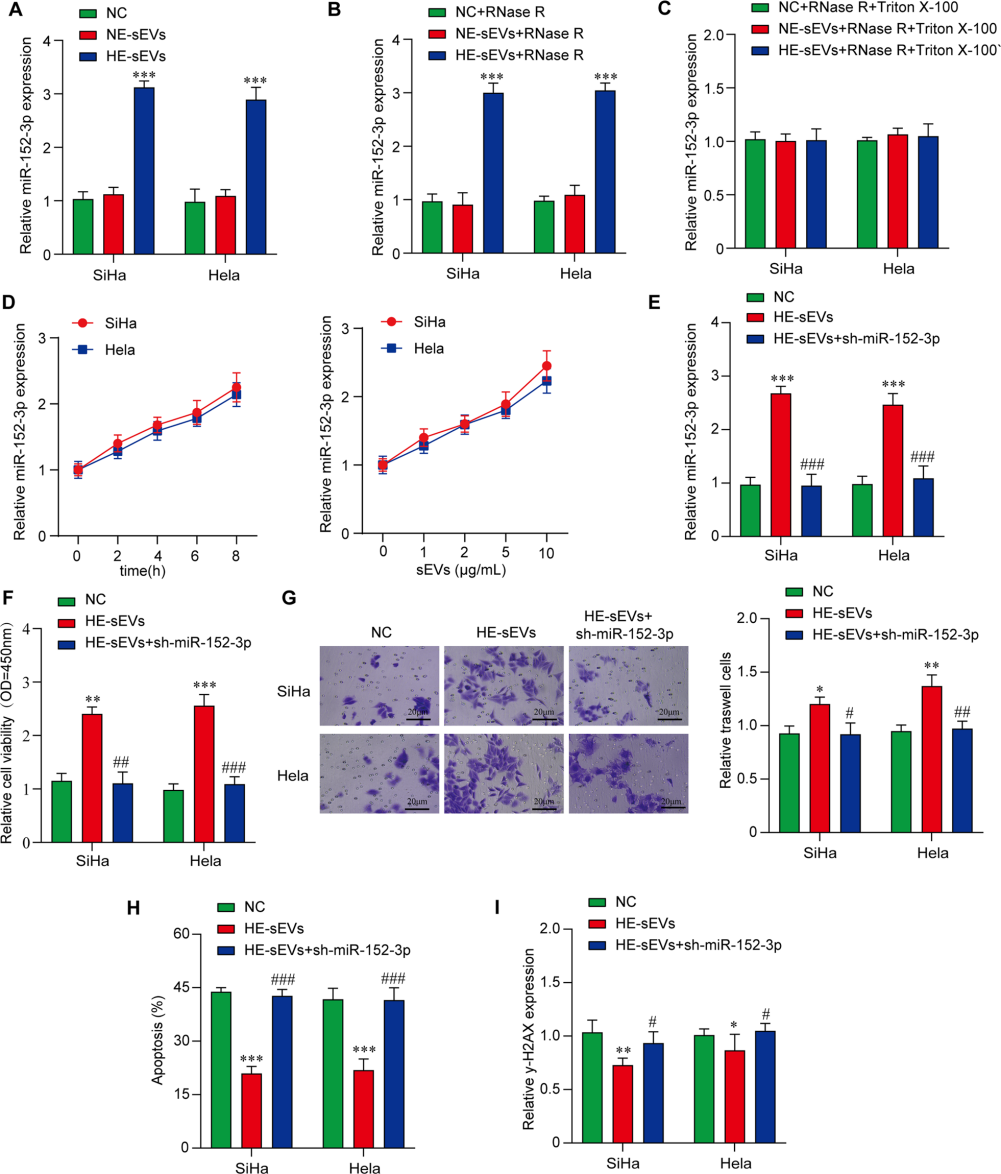
MiR-152-3p acts as an effector cargo molecule in hypoxic cells-derives sEVs in CC radiation response. **A** The expression levels of miR-152-3p in SiHa and Hela CC cells after HE-sEVs or NE-sEVs treatment was examined by qRT-PCR. ***P < 0.001 versus negative control CC or NE-sEV cells. **B** The different expressions of sEV miR-152-3p in SiHa and Hela CC cells treated with NC + RNase R (0.1 mg/mL), HE-sEVs + RNase R or NE-sEVs + RNase R were shown by qRT-PCR. ***P < 0.001 versus CC cells sEVs treated with RNase R. **C** qRT-PCR was used to detect the expression of miR-152-3p in sEVs treated with different oxygen concentrations and destroyed with TritonX-100 (0.3%) and RNase R (0.1 mg/ml). **D** The level of miR-152-3p in SiHa and Hela cells is correlated with the time and dose substitution of HE-sEVs treatment. **E** Inhibition of miR-152-3p by sh-miR-152-3p transfection in HE-sEVs was confirmed by qRT-PCR. ***P < 0.001 versus NC group, ^*###*^*P* < 0.001 versus HE-sEVs group. **F** Effects of different treatments on proliferation of SiHa and HeLa cells was measured by CCK-8 assay. **P < 0.01, ***P < 0.001 versus NC group, ^*##*^*P* < *0.01, *^*###*^*P* < 0.001 versus H-EV group. **G** Transwell assay of SiHa and Hela cells with different treatments was conducted, and the representative images were shown. *P < 0.05, **P < 0.01 versus NC group, ^*#*^*P* < 0.05, ^*##*^*P* < 0.01 vs HE-sEVs group. **H** The effects of different treatments on apoptosis of SiHa and HeLa cells were analyzed by flow cytometry. ***P < 0.001 versus NC group, ^*###*^*P* < 0.001 versus HE-EVs group. **I** The level of γ-H2AX expression in SiHa and Hela cells with different treatments was quantified. All irradiation treatment doses used were 4 Gy. *P < 0.05, **P < 0.01 versus NC group, ^*#*^*P* < 0.05 versus HE-sEVs group

Our results showed that the expression of miR-152-3p in HE-sEV treatment group was significantly higher than that in PBS control or NE-sEV treatment group, but no significant changes were observed between PBS control group and NE-sEV treatment group in both SiHa and Hela cell lines (Fig. 3A). Using RNase R to destroy the residual RNA outside sEVs to eliminate its interference to the results, we found similar result that the expression of miR-152-3p in HE-sEV treatment group was significantly higher than that in PBS control or NE-sEV treatment group. Our result verified that miR-152-3p was derived from hypoxia cells-derived sEVs (Fig. 3B). We then used Triton X-100 to destroy sEVs followed by RNase R to destroy the RNAs encapsulated in sEVs, and the result showed a reversion of the increased level of miR-152-3p (Fig. 3C). Moreover, the increased expression level of miR-152-3p was found in a time-dependent and dose-dependent manner after culturing cells with HE-sEVs (Fig. 3D). These data suggest that miR-152-3p was wrapped in HE-sEVs derived CC cells to play a role. To further explore the function of HE-sEV miR-152-3p on CC cell radioresistance, HE-sEV miR-152-3p was suppressed by transfecting CC cell lines with sh-miR-152-3p (Fig. 3E). After RT, the cell viability and invasion ability were significantly reduced in both SiHa and Hela cell lines incubated with sh-miR-152-3p transfected HE-sEVs compared with HE-sEVs (without transfection) incubations (Fig. 3F, G). The inhibition of HE-sEV miR-152-3p was found to be associated with increased Annexin V and γ-H2AX expressions in both SiHa and Hela cell lines compared with HE-sEVs (without transfection) incubations, demonstrating this inhibition induces more apoptosis and DNA damage and reverses radioresistance induced by hypoxia treatment (Fig. 3H, I). Our findings confirm the importance of sEV miR-152-3p induced by hypoxic environment for CC radiation response.

### KLF15 is the downstream target of miR-152-3p

**Fig. 4:**
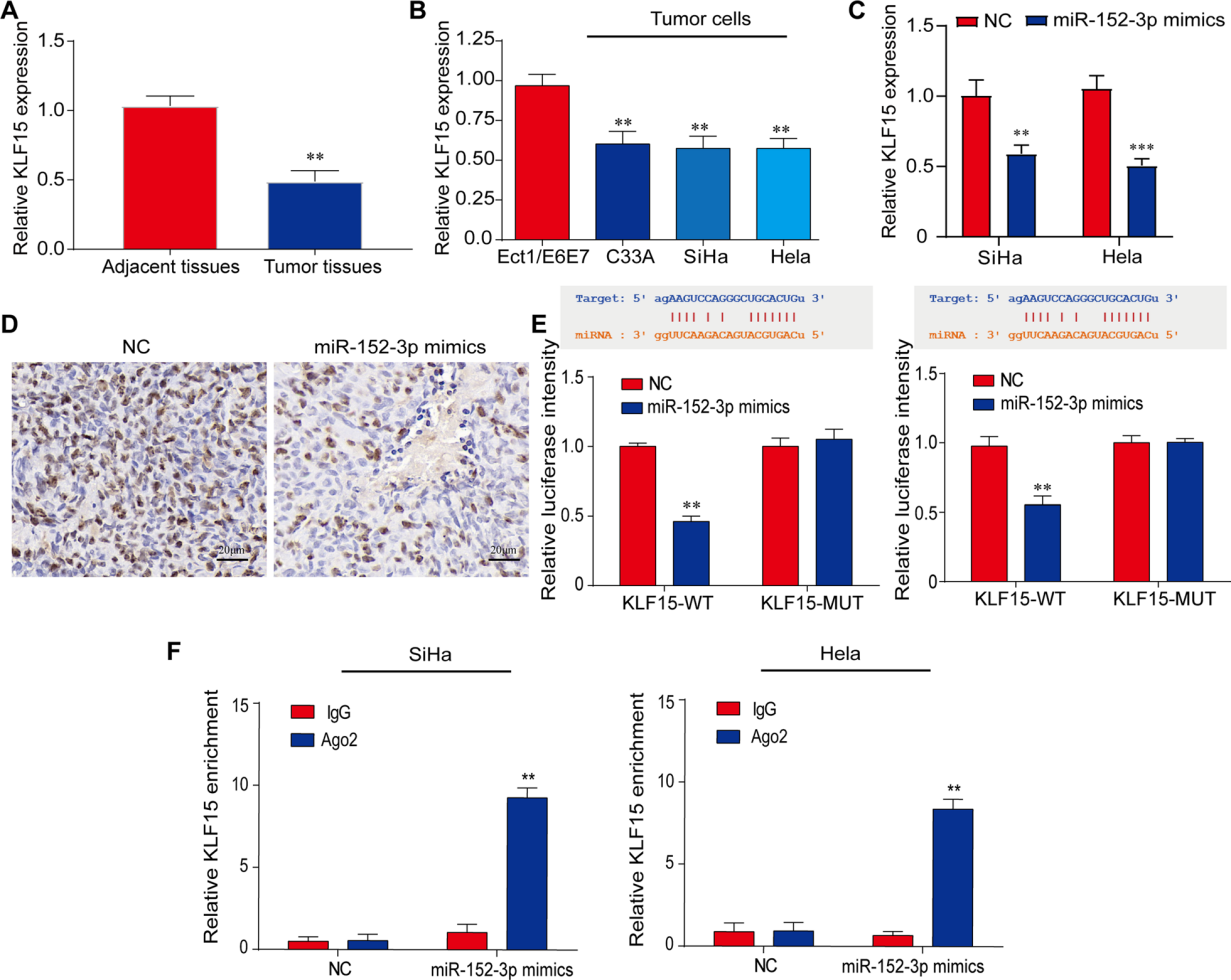
KLF15 is a downstream target of miR-152-3p in CC cells. **A** The reduced expression of KLF15 was found in cervical tumor tissues compared with adjacent tissues from CC patients by qRT-PCR (n = 20 in each group), **P < 0.01 versus adjacent tissues. **B** The reduced expression of KLF15 was shown in CC cells (C33A, SiHa and Hela) compared with normal cervical epithelial cell (Ect1/E6E7) by qRT-PCR. **P < 0.01 versus Ect1/E6E7 cells. **C** The expression of KLF15 in SiHa and Hela cells was reduced after treatment with miR-152-3p mimics compared with untreated control by qRT-PCR. **P < 0.01, ***P < 0.001 versus NC group. **D** The expression of KLF15 was reduced in animal tumor xenografts treated with miR-152-3p mimics compared with control. Representative images are shown for KLF15 expression in treated and control groups by IHC. The brown indicates positive staining while the blue indicates nuclei, scale bar: 20 μm. **E** The direct binding of miR-152-3p to the 3'-UTR region of KLF15 was shown by dual luciferase report assay. The significant difference was found between KLF15-WT and KLF15-MUT after miR-152-3p mimics treatments **P < 0.01 versus NC group. **F** RIP assay was shown the binding status between miR-152-3p and KLF15 in SiHa and Hela cells, confirming the miR-152-3p specifically binding KLF15. **P < 0.01 versus miR-152-3p mimics IgG group

To investigate the mechanism of miR-152-3p in the regulation of CC radioresistance and identify the downstream targets of miR-152-3p, we first screened significantly down-regulated mRNAs from the TCGA database and cross-selected candidates with the target genes of miR-152-3p. We found that miR-152-3p was associated with its downstream targets KLF15, KLF2, KLF8 and KLF9 (Additional file 1: Fig. S2A-C). After validation through GEPIA and Starbase databases, KLF15 was found to have the most significant difference between CC tissues and control tissues (Additional file 1: Fig. S 2D, E). In comparison of human CC tissues with adjacent control tissues, the low-level expression of KLF15 was found in CC tissues (Fig. 4A). Similarly, in comparison of human CC cell lines with a cervical control cell line, the low-level expression of KLF15 was also found in CC cell lines (Fig. 4B). qRT-PCR analysis showed that KLF15 level was decreased in CC cells with miR-152-3p overexpression compared with control cells (Fig. 4C). In consistence, IHC results showed that the number of KLF15-positive cells in animal xenograft tissues significantly decreased in the treatment with miR-152-3p mimics compared with control (Fig. 4D). Double luciferase report analysis showed that the relative luciferase activity of KLF15-WT in SiHa and Hela cells was limited after co-transfection with miR-152-3p mimics, but the luciferase activity of KLF15-mutant plasmid did not change, indicating that KLF15 was the direct target of miR-152-3p (Fig. 4E). RIP results indicated that KLF15 was upregulated in miR-152-3p mimics and down-regulated in miR-152-3p inhibitor in SiHa and Hela CC cell lines (Fig. 4F). These results suggest that KLF15 is a direct target of miR-152-3p.

### miR-152-3p regulates CC radioresistance by targeting KLF15

**Fig. 5:**
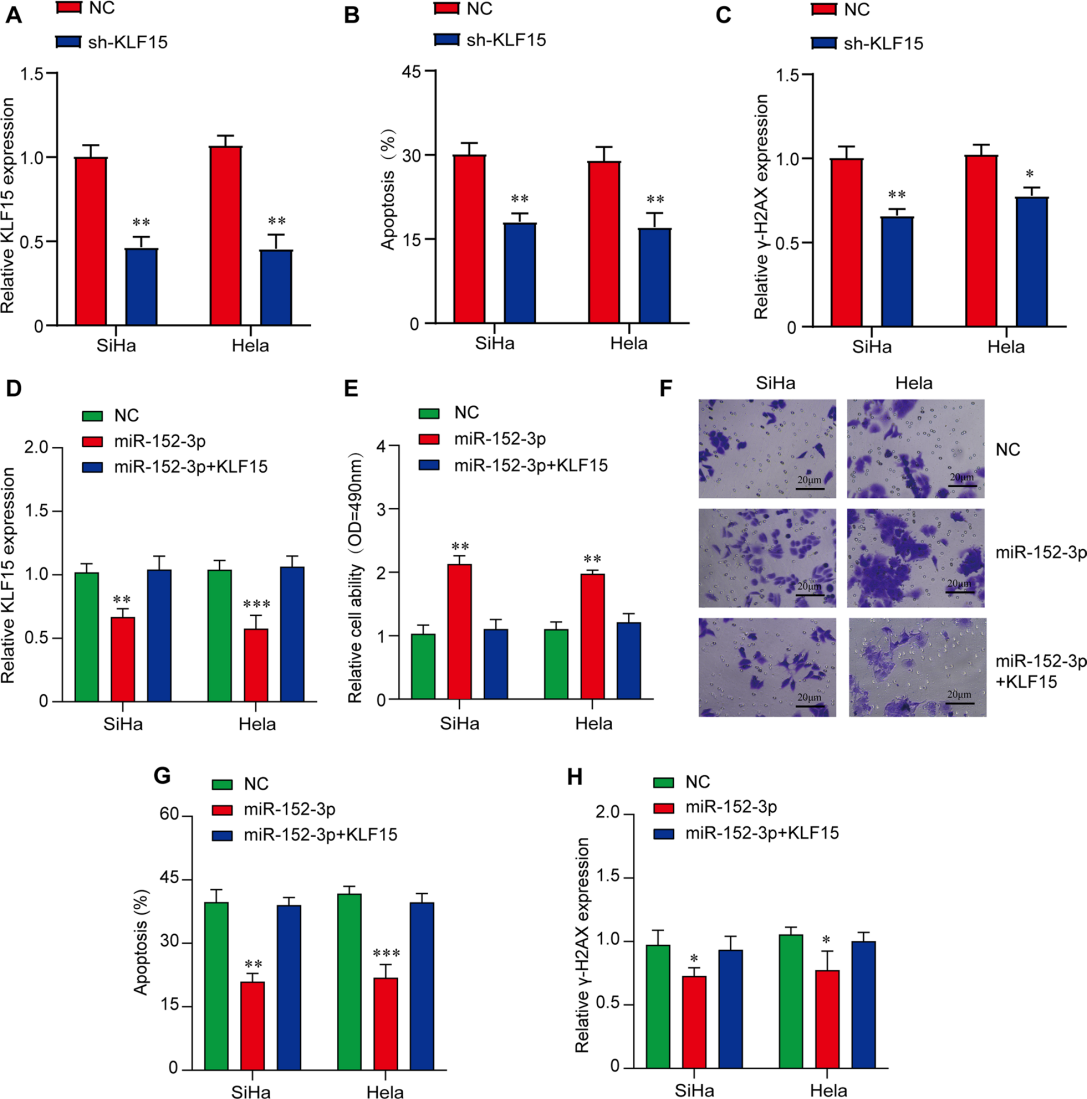
MiR-152-3p exerts it’s radioresistant effect on CC cells by targeting KLF15. **A** qRT-PCR analysis verified the expression of KLF15 knockdown by transfecting shRNA in SiHa and Hela cells. **P < 0.01 versus NC group. **B** The apoptosis in KLF15 knockdown CC cells was significantly reduced compared with control after 72 h of radiotherapy by flow cytometry. **P < 0.01 versus NC group. **C** The expression of γ-H2AX after transfection in KLF15 knockdown CC cells was significantly reduced compared with control by qRT-PCR. *P < 0.05 and **P < 0.01 versus NC group. **D** qRT-PCR verified the expression of KLF15 in SiHa and Hela cells after different transfections as indicated and significant difference was found between miR-152-3p overexpression group and NC group. **P < 0.01 and ***P < 0.001 versus NC group. **E** The proliferation of SiHa and Hela cells after different transfections was shown by CCK-8 assay and significant difference was found between miR-152-3p overexpression group and NC group. **P < 0.01 versus NC group. **F** Cell migration in SiHa and Hela cells was measured by transwell assay after different transfections. Representative images are shown for NC, miR152-3p and miR152-3p + KLF15 from 3 independent experiments (n = 3), scale bar: 20 μm. **G** The apoptosis assay of SiHa and Hela cells after different transfections was shown by flow cytometry, and the significant difference was found between miR-152-3p overexpression group and NC group. **P < 0.01 and ***P < 0.001 versus NC group. **H** The expression of γ-H2AX in SiHa and Hela cells after different transfections was quantified by qRT-PCR and the significant difference was found between miR-152-3p overexpression group and NC group. *P < 0.05 versus NC group. *P < 0.05 and **P < 0.01 versus NC group

To determine the biological role of KLF15, we constructed a lentiviral vector of KLF15 inhibitor and transfected it into SiHa and Hela cells (Fig. 5A). The results showed that silencing KLF15 significantly reduced apoptosis after IR treatment (Fig. 5B). In addition, inhibition of KLF15 expression also reduced DNA damage caused by IR (Fig. 5C). These findings indicated that KLF15 was a tumor suppressor and played an important role in cell damage after radiation treatment. In addition, we overexpressed KLF15 in SiHa and Hela cells by transfection of miR-152-3p to examine whether KLF15 was the substrate that affected cell radiosensitivity. The results showed that the overexpression of KLF15 reversed the increased proliferation and migration of CC cells caused by miR-152-3p overexpression (Fig. 5D, F). In addition, KLF15 expression rescinded the changes in key protein levels during miR-152-3p induced apoptosis and DNA damage repair (Fig. 5G, H). These results suggest that miR-152-3p promotes radioresistance by directly targeting KLF15.

### Expression of plasma sEV miR-152-3p and KLF15 is associated with CC prognosis in RT

**Fig. 6:**
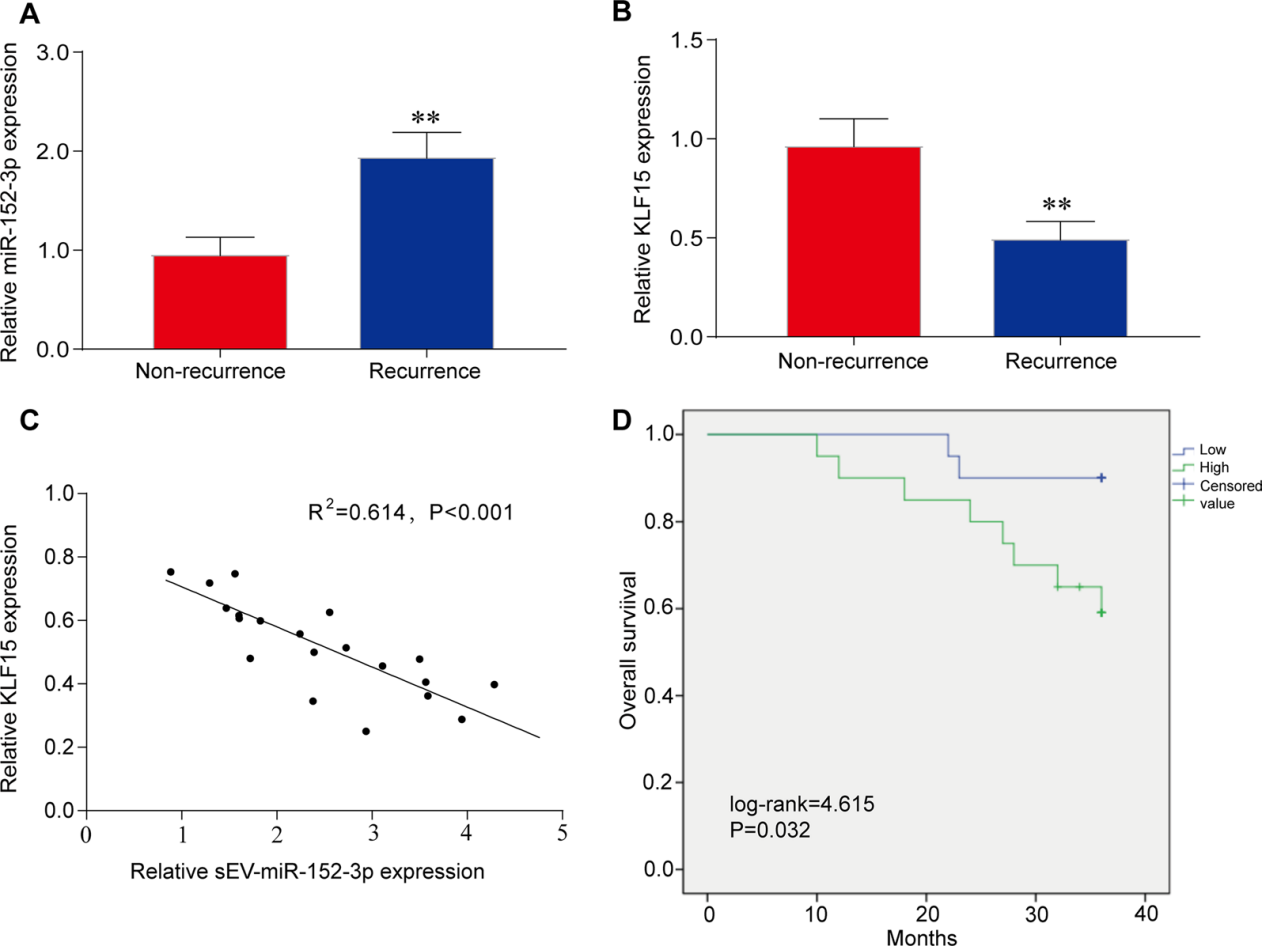
Expression of plasma sEV miR-152-3p and KLF15, and its prognostic value. **A** The level of sEV miR-152-3p in CC patients with or without recurrence after radiotherapy was analyzed by qRT-PCR (n = 20 in each group), indicating increased sEV miR-152-3p was found in recurrent CC patients compared with non-recurrent CC patients. **P < 0.01 vs non-recurrence. **B** The expression of KLF15 in the above patients (n = 20 in each group) was shown by qRT-PCR verifies, indicating reduced sEV KLF15 was found in recurrent CC patients compared with non-recurrent CC patients. **P < 0.01 vs non-recurrence. **C** Correlation analysis between miR-152-3p and KLF15 in the same patients was performed qRT-PCR, the expression of KLF15 mRNA in CC tissues of these patients was negatively correlated with the level of plasma sEV miR-152-3p. **P < 0.01 vs non-recurrence. **D** Kaplan–Meier survival rate analysis of the overall survival rate of these patients by miR-152-3p expression level in tissues was analyzed by Kaplan–Meier analysis. **P < 0.01 vs non-recurrence

The expression of plasma sEV miR-152-3p and KLF15 was analyzed in 20 CC patients who received explicit RT. A high level of sEV miR-152-3p expression in patients who relapsed within 3 years after RT was identified (Fig. 6A). Further detection of KLF15 expression by qRT-PCR showed that sEV KLF15 expression was significantly lower in the recurrence patients than that in the non-recurrence group (Fig. 6B). In addition, qPCR analysis confirmed that the expression of KLF15 mRNA in CC tissues of these patients was negatively correlated with the level of plasma sEV miR-152-3p (R^2^ = 0.614, *P* < 0.001) (Fig. 6C). Furthermore, patients with lower miR-152-3p level had worse overall survivals by Kaplan–Meier analysis (Fig. 6D). Taken together, our findings indicate that both sEV miR-152-3p and KLF15 mRNA markers can be used to predict CC radioresistance.

## Discussion

RT is widely recognized in the treatment of CC with effective consequences. However, the gradual increase of radioresistance is a major problem limiting the clinical treatment efficacy of CC patients [27]. Therefore, further research investigating the molecular mechanism of radioresistance to increase CC cell sensitivity is an important step for a better curative effect to improve the prognosis and survival of CC patients. Hypoxia has been found to be one of the important factors in radioresistance [5, 7]. The mechanism of radioresistance of hypoxic cells has been extensively studied in CC such as in cancer stem cell-like phenotype and glucose metabolism [28, 29]. However, the TME and cell communication under HE has not been elucidated yet in CC, which is still needed to be further explored in this area [30].

EVs derived from cancer cells are important mediators in cell communication, which play various roles in regulating cell proliferation, progression, drug resistance, immune escape and metastasis [31]. Several lines of evidence demonstrate that increased number of EVs is produced in tumor hypoxic environment [32]. In addition, recent reports also showed EV cargos from hypoxic cells were transferred to normoxic cells through internalization, so the cargo molecules contained in the EVs were also transferable to normoxic cells [19]. In this study, we tested NTA, total proteins and RNAs to verify that the HE led to an increased number of sEVs secreted by CC cells. Accumulating reports show that sEV miRNAs contribute to the cancer progression with implications for both diagnosis and treatment [33]. sEV cargos from hypoxic cells could be transferred to normoxic cells to play a biological role [19]. Tumor cell-derived sEV miRNAs from hypoxia usually further aggravate the malignant transformation of normoxic cells. In the current study, we demonstrate that the EVs secreted by hypoxic CC cells are higher than normoxic cells, and EVs derived from hypoxic CC cells can reduce DNA damage induced by RT.

The in vivo and in vitro results of this study suggest that after incubation with hypoxic-derived sEVs, the proliferation and radioresistance of CC cells were significantly increased, indicating that they have a cancer-promoting effect. In the meanwhile, we found that miR-152-3p was highly expressed in hypoxic CC cell-derived sEV. The expression of miR-152-3p was significantly different in various cancer tissues and cells. For example, the upregulation of miR-152-3p was reported to play an anti-tumor role by negatively regulating PI3KCA to inhibit the proliferation of breast cancer cell HCC1806 [34]. However, miR-152-3p was found to be overexpressed in chronic myeloid leukemia (CML), which promoted K562 cell proliferation by inhibiting P27, thereby increasing its malignant potential [35]. The data of this study showed that the expression of sEV miR-152-3p was increased under HE, which is essential for HE-sEV-induced radioresistance for CC. Using bioinformatics analysis, we found KLF15 was a direct target of miR-152-3p that contributed to CC radioresistance. The KLF family is engaged in numerous cell biological processes, including proliferation and apoptosis, according to earlier research [36]. The level of KLF15 in ovarian cancer tissues and cells is lower than normal. Overexpression of KLF15 suppressed the proliferation and migration of ovarian cancer cells [37]. In addition, low KLF15 expression level indicates a poor prognosis of gastric cancer patients [38]. However, the role of KLF15 in CC has not been reported. We explored for the first time the impact of KLF15 in proliferation, apoptosis and DNA damage for CC. KLF15 overexpression reverses the inhibition of apoptosis and DNA damage by miR-152-3p in CC cells. KLF15 improves radiosensitivity by enhancing apoptosis of CC cells. sEV miR-152-3p and KLF15 are potential therapeutic targets to improve CC radiosensitivity.

Finally, we verified the expression of plasma sEVs miR-152-3p increased in CC patients who relapsed after RT. In addition, the levels of plasma sEV miR-152-3p and KLF15 have shown encouraging results in predicting CC prognosis in RT. At present, liquid biopsy of EV biomarkers such as miRNAs, mRNAs and proteins is a new developing area for the early diagnosis, predicting prognosis and monitoring cancer progression [39, 40]. Since increased sEV miR-152-3p and reduced sEV KLF15 were found in cervical patients’ plasma after recurrence after RT, these two EV markers hold promise for liquid biopsy to predict CC radioresistance or recurrence after RT. However, more clinical samples are needed to further validate their values [41].

## Conclusion

In summary, our findings firstly demonstrate that sEVs from hypoxic cells enhance the CC radioresistance by transferring miR-152-3p. The mechanism of sEV miR-152-3p involved is associated with blocking IR-induced apoptosis and DNA repair by targeting KLF15. In addition, increased plasma sEV miR-152-3p and reduced KLF15 protein have shown promise in predicting CC prognosis after RT. Our results suggest sEV miR-152-3p and KLF15 are useful biomarkers for predicting CC radiation response as well as potential therapeutic targets for developing novel EV-based targeted therapy to improve CC RT and overcome radioresistance.

### Supplementary Information

**Additional file 1: Fig. S1**. Verification of sEVs after treatment with different oxygen concentrations (HE and NE). (A, B) The concentration of sEVs secreted by cells in different treatment groups was measured by NTA. *P<0.05 vs. CC cells treated with normoxia. (C) The morphology of sEV was observed by transmission electron microscopy (TEM). (D, E) The expression of sEV markers in different CC cells was detected by western blot analysis and quantified. **P<0.01 vs. CC cells treated with normoxia. (F) The expression of HIF-1α after different treatments was detected by western blot analysis and quantified. **P<0.01 vs. CC cells treated with normoxia. (G) Immunofluorescence detects the entry of sEVs into CC cells after PKH67 labeling, red indicates sEVs and blue indicates cell nucleus, scale bar: 20 μm.

**Additional file 2: Fig. S2**. Target gene screening of miR-152-3p. (A, B) Heat map and volcano map show the differentially expressed mRNAs in the TCGA database. (C) Venn diagram is used to screen target mRNAs. (D) GEPIA database verifies the differential expression of KLF15 in CC tissues and normal cervical tissues. (E) The Starbase database (ENCORI) verifies the correlation between miR-152-3p and KLF15 in CC tissues.

## Data Availability

Any individual or group may contact the corresponding author to obtain relevant research data.

## Abbreviations

ANOVA: Analysis of variance
Bcl-2: B-cell lymphoma-2
CD: Cluster of differentiation
DNA: Deoxyribonucleic acid
EVs: Extracellular vesicles
GAPDH: Glyceraldehyde-3-phosphate dehydrogenase
GEO: Gene expression omnibus
GEPIA: Gene expression profiling interactive analysis
H2AX: H2A.X variant histone
HE: Hematoxylin and Eosin
HE-EV: Hypoxia environment secreted extracellular vesicle
HIF-1α: Hypoxia-inducible factor 1α
HRP: Horseradish peroxidase
IgG: Immunoglobulin G
IHC: Immunohistochemistry
IR: Ionizing radiation
KLF15: Kruppel-like factor 15
miRNA: MicroRNA
NCBI: National Center for Biotechnology Information
NE-EV: Normoxia environment secreted extracellular vesicle
NTA: Nanoparticle tracking analysis
P21: Cyclin-dependent kinase inhibitor 1A
P56: Cyclin-dependent kinase like 2
PBS: Phosphate buffered saline
PCR: Polymerase chain reaction
PI: Propidium iodide
PVDF: Polyvinylidene fluoride
qRT-PCR: Quantitative reverse transcription polymerase chain reaction
RIPA: Radio-immunoprecipitation assay
RNA: Ribonucleic acid
SDS–PAGE: Sodium dodecyl sulfate–polyacrylamide gel electrophoresis
TCGA: The cancer genome atlas
TME: Tumor microenvironment
UTR: Untranslated regions
