# Supplementary material for: Hypoxic tumor cell-derived small extracellular vesicle miR-152-3p promotes cervical cancer radioresistance through KLF15 protein
Source: Radiat Oncol. 2023 Nov 7;18:183. doi: 10.1186/s13014-023-02369-3 (PMC10631204; doi:10.1186/s13014-023-02369-3)
Supplement: Supplementary file 1 — Additional file 1: Fig. S1. Verification of sEVs after treatment with different oxygen concentrations (HE and NE). (A, B) The concentration of sEVs secreted by cells in different treatment groups was measured by NTA. *P<0.05 vs. CC cells treated with normoxia. (C) The morphology of sEV was observed by transmission electron microscopy (TEM). (D, E) The expression of sEV markers in different CC cells was detected by western blot analysis and quantified. **P<0.01 vs. CC cells treated with normoxia. (F) The expression of HIF-1α after different treatments was detected by western blot analysis and quantified. **P<0.01 vs. CC cells treated with normoxia. (G) Immunofluorescence detects the entry of sEVs into CC cells after PKH67 labeling, red indicates sEVs and blue indicates cell nucleus, scale bar: 20 μm. [file 13014_2023_2369_MOESM1_ESM.docx]

**Supplementary figures**

**Supplementary Figure 1. Verification of sEVs after treatment with different oxygen concentrations (HE and NE).**

**
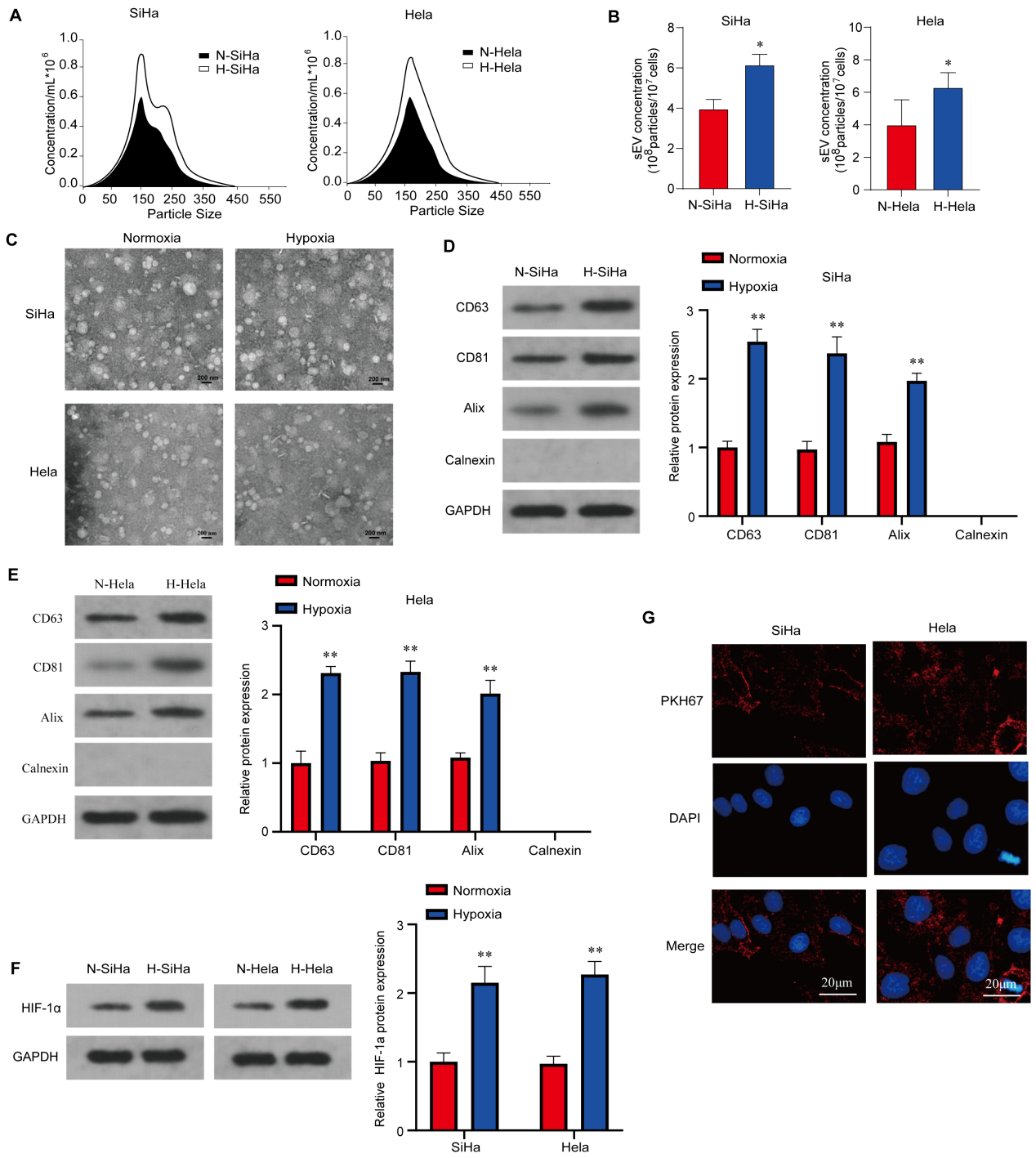
**

**Supplementary Figure 2. Target gene screening of miR-152-3p**

**
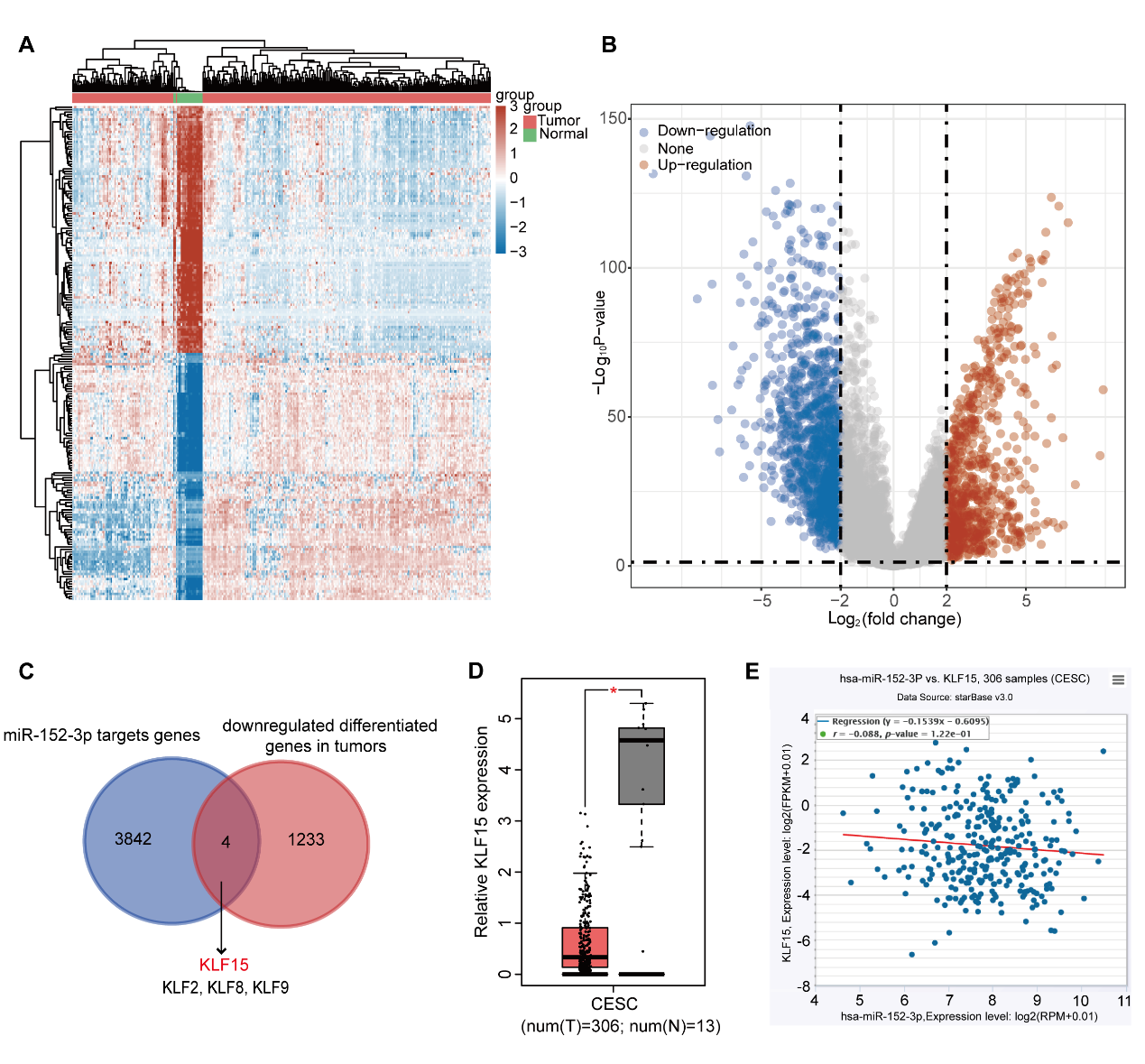
**

**Supplementary tables**

**Supplementary table 1. Plasmids of miR-152-3p mimic, inhibitor and controls**

|  | Primer Sequence |
| --- | --- |
| miR-NC inhibitor | 5ʹ-UCGCUUGGUGCAGGUCGGGAA-3ʹ |
| miR-152-3p inhibitor | 5ʹ-CCAAGUUCUGUCAUGCACUGA-3ʹ |
| miR-NC mimic | 5ʹ-GGAACUUAGCCACUGUGAAUU-3ʹ |
| miR-152-3p mimic | 5ʹ-UCAGUGCAUGACAGAACUUGG-3ʹ |

**Supplementary table 2. Primer sequence**

|  | Primer Sequence |
| --- | --- |
| miR-152-3p forward | 5'- ACACTCCAGCTGGGTCAGTGCATGACAG -3 ' |
| miR-152-3p reverse | 5'- CTCAACTGGTGTCGTGGAGTCGGCAATTCAGTTGAGCCAAGTT -3' |
| U6 forward | 5’-CTCGCTTCGGCAGCACATA-3’ |
| U6 reverse | 5’-AACGATTCACGAATTTGCGT-3’ |
| KLF15 forward | 5’- CCA AAA GCA GCC ACC TCA AG -3’ |
| KLF15 reverse | 5’- GAC ACT GGT ACG GCT TCA CA -3’ |
| GAPDH forward | 5’-GAAGGTGAAGGTCGGAGTC-3’ |
| GAPDH reverse | 5’-GAAGATGGTGATGGGATTTC-3’ |

**Supplementary table 3. Antibodies for western blotting**

| Name | Dilution and company |
| --- | --- |
| γ-H2AX | 1:1000, Abcam, Hong Kong, China |
| p-DNApcks | 1:1000, Abcam, Hong Kong, China |
| KLF15 | 1:1000, Abcam, Hong Kong, China |
| CD63 | 1:1000, Abcam, Hong Kong, China |
| CD81 | 1:1000, Abcam, Hong Kong, China |
| Aliex | 1:1000, Abcam, Hong Kong, China |
| calnexin | 1:1000, Abcam, Hong Kong, China |
| GAPDH | 1:5000, Beyotime, Shanghai, China |
