# Supplementary material for: Hypoxic tumor cell-derived small extracellular vesicle miR-152-3p promotes cervical cancer radioresistance through KLF15 protein
Source: Radiat Oncol. 2023 Nov 7;18:183. doi: 10.1186/s13014-023-02369-3 (PMC10631204; doi:10.1186/s13014-023-02369-3)
Supplement: Supplementary file 2 — Additional file 2: Fig. S2. Target gene screening of miR-152-3p. (A, B) Heat map and volcano map show the differentially expressed mRNAs in the TCGA database. (C) Venn diagram is used to screen target mRNAs. (D) GEPIA database verifies the differential expression of KLF15 in CC tissues and normal cervical tissues. (E) The Starbase database (ENCORI) verifies the correlation between miR-152-3p and KLF15 in CC tissues. [file 13014_2023_2369_MOESM2_ESM.docx]

**Supplementary methods**

**Transmission electron microscope (TEM)**

TEM was used to determine the morphology of sEV samples. sEV suspension was fixed in 4% paraformaldehyde for 1 h, and 4 μl EV suspension was then applied to 400 mesh copper mesh Formvar coated carbon stabilized grids for 4-5 min, and dried with filter paper. Negative staining was performed for 2 min in the same approach and imaging was obtained with TEM (JEM-1400, Hitachi, Shiga, Japan).

**Nanoparticle tracking analysis (NTA)**

The size distribution and concentration of sEVs were detected using the NanoSight NS300 system (Malvern Instruments, Malvern, UK). In short, the separated sEVs were diluted with 1:500 or 1:1000 in freshly 0.22 μm filtered PBS and loaded into the detection room with a syringe. The highly sensitive sCMOS camera, fast video capture and particle tracking software (NanoSight, Amesbury, UK) were used to identify and track the center of each particle under Brownian motion. The original particle concentration of the separator was calculated according to the measured concentration and dilution coefficient.

**Cell proliferation**

Proliferating capacity of transfected cervical cancer cell lines SiHa and Hela and control cell line Ect1/E6E7 was detected by the Cell Counting Kit-8 (DOJINDO, Japan) according to the instructions. Briefly, 2 × 10^4^ cells in 0.1 mL complete medium were seeded in 96-well plates and incubated at 37 °C for 24 h and humidified 5% CO_2_, and then the cell numbers of each well were analyzed by a microplate reader (BIO-RAD Laboratories, Philadelphia, PA, USA) with the 450 nm absorbance. Cell proliferation rate is presented as the fold change of absorbance.

**Invasion assay**

Invasive ability of transfected CC cells was assessed by commercial matrigel and transwell chambers (BD Bioscience, CA, US). Briefly, the day (12 h) before the invasion experiment, the diluted matrigel (BD Biosciences, CA, USA) was coated on the upper chamber of the transwell plate and stored at 37 ℃ o/n. 1×10^5^ cells in 500 μL serum-free MEM medium were added to each transwell insert, respectively. Then, cells were inserted into a 24-well plate with 500 μL MEM containing 10% FBS. After incubation at 37 ℃ in humidified 5% CO_2_ for 24 h, the cells in the upper chamber were removed. After paraformaldehyde fixation and staining with 0.5% crystal violet, the cells were counted under an inverted optical microscope (Leica, Germany).

**Immunohistochemistry (IHC)**

Paraffin sections were dewaxed and rehydrated, blocked with 5% skimmed milk powder for 1 h, and then incubated with the primary antibody Ki67 at 4 ℃ o/n. The rabbit biotinylated IgG second antibody was incubated at room temperature for 45 min, then tissue sections were stained with 3, 3′-diaminobenzidine and hematoxylin in succession. The image was recorded through a light microscope (Nexcope NE900, China).

The staining intensity (0–3) was assessed using a light microscopy (Leica, Germany). The assessment criteria is as followed: 0 (negative, < 25%), 1 (weak, 25–50%), 2 (moderate, 50–75%), 3 (strong, > 75%) of the tumor cells stained. Blind scoring was performed independently by three experienced observers (LC, NL and YL), and the average of grades was recorded.
